# Molecular dynamics simulations of site point mutations in the TPR domain of cyclophilin 40 identify conformational states with distinct dynamic and enzymatic properties

**DOI:** 10.1063/1.5019457

**Published:** 2018-04-09

**Authors:** Mert Gur, Elizabeth A. Blackburn, Jia Ning, Vikram Narayan, Kathryn L. Ball, Malcolm D. Walkinshaw, Burak Erman

**Affiliations:** 1 Department of Mechanical Engineering, Faculty of Mechanical Engineering, Istanbul Technical University (ITU), Suite 445 İnönü Caddesi, No. 65 Gümüşsuyu, 34437 Beyoğlu, Istanbul, Turkey; 2 Centre for Translational and Chemical Biology, School of Biological Sciences, University of Edinburgh, Edinburgh EH9 3FF, United Kingdom; 3 Institute of Genetics and Molecular Medicine, University of Edinburgh, Edinburgh EH4 2XU, United Kingdom; 4 Department of Chemical and Biological Engineering, Koc University College of Engineering, Eng 146 Rumeli Feneri Yolu, 34450 Sarıyer, Istanbul, Turkey

## Abstract

Cyclophilin 40 (Cyp40) is a member of the immunophilin family that acts as a peptidyl-prolyl-isomerase enzyme and binds to the heat shock protein 90 (Hsp90). Its structure comprises an N-terminal cyclophilin domain and a C-terminal tetratricopeptide (TPR) domain. Cyp40 is overexpressed in prostate cancer and certain T-cell lymphomas. The groove for Hsp90 binding on the TPR domain includes residues Lys227 and Lys308, referred to as the carboxylate clamp, and is essential for Cyp40-Hsp90 binding. In this study, the effect of two mutations, K227A and K308A, and their combinative mutant was investigated by performing a total of 5.76 *μ*s of all-atom molecular dynamics (MD) simulations in explicit solvent. All simulations, except the K308A mutant, were found to adopt two distinct (extended or compact) conformers defined by different cyclophilin-TPR interdomain distances. The K308A mutant was only observed in the extended form which is observed in the Cyp40 X-ray structure. The wild-type, K227A, and combined mutant also showed bimodal distributions. The experimental melting temperature, T_m_, values of the mutants correlate with the degree of compactness with the K308A extended mutant having a marginally lower melting temperature. Another novel measure of compactness determined from the MD data, the “coordination shell volume,” also shows a direct correlation with T_m_. In addition, the MD simulations show an allosteric effect with the mutations in the remote TPR domain having a pronounced effect on the molecular motions of the enzymatic cyclophilin domain which helps rationalise the experimentally observed increase in enzyme activity measured for all three mutations.

## I. INTRODUCTION

Cyclophilin 40 (also known as Cyp40 or PPID) is a two-domain protein member of the immunophilin family. In common with most of the other 20 cyclophilins in the human genome, Cyp40 acts as a peptidyl-prolyl-isomerase (PPIase) enzyme which speeds up rotation around the peptidyl prolyl amide bond resulting in more efficient folding of peptide chains.^1^ Cyp40 is a potential therapeutic agent in protein misfolding diseases, as it interacts at sites rich in proline residues and can unravel neurotoxic amyloids composed of either Tau or α-synuclein.^2^ This effect appears to be isomerase dependent and specific to Cyp40 within the tetratricopeptide (TPR) domain prolyl-isomerases. Cyp40 is unique in the cyclophilin family of immunophilins in having a TPR domain which anchors the protein to heat shock protein 90 (Hsp90). The Hsp90-Cyp40 co-chaperone complex is implicated in the folding and release of Lck and Fes tyrosine kinases^3^ and also regulates androgen-dependent cell proliferation.^4^ Cyp40 is overexpressed in prostate cancer^4^ and certain T-cell lymphomas,^5^ suggesting that inhibition of complex formation with Hsp90 may also be therapeutically useful.

**FIG. 1.**
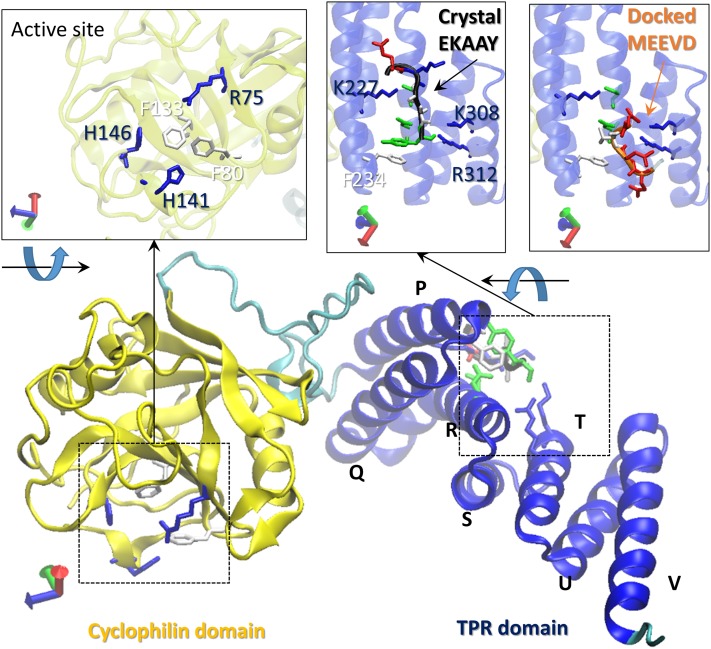
Cyp40 architecture with helix numbering. The crystal structure of Cyp40 (PDB code 1IHG) is shown. The N-terminal cyclophilin domain (residues 1–183) is shown in yellow, whereas the α-helical C-terminal TPR domain (residues 214-362) is shown in blue. The active site residues (Arg75, Phe80, Phe133, His141, His146) are shown in the upper left panel. The upper middle panel shows the crystal contacts with the C-terminal EKKAY stretch of the neighboring Cyp40. The upper right panel shows the Cyp40-MEEVD peptide interaction obtained via docking.^10^

The two domain structure of Cyp40 (PDB code 1IHG)^6^ is shown in the lower panel of Fig. 1. The protein is composed of an N-terminal cyclophilin domain (residues 1–183, shown in yellow) and an α-helical C-terminal TPR domain (residues 214-362, shown in blue). The active site residues (Arg75, Phe80, Phe133, His141, His146) shown in the upper left panel are conserved among almost all cyclophilins with PPIase activity. Cyp40 belongs to a subgroup of cyclophilins containing an inserted, so-called divergent loop, in the cyclophilin domain, 60PTTGKPLH67. This is not found in the archetypal cyclophilin A structures and has been suggested to provide specificity in binding.^7^ The cyclophilin and TPR domains are connected by a highly charged linker (residues L184-L213), with over 30% are aspartates or glutamates, forming a number of salt bridges with both the TPR and cyclophilin domains. These salt bridges are important in determining the relative orientations of the cyclophilin and TPR domain. Domain orientation is thought to be important for substrate recognition is FKBP51 and FKBP52, large immunophilins that also bind Hsp90 through a TPR domain and compete with Cyp40 (Ref. 23).

The TPR domain comprises 7 helices. The first two helices, P (V216-K235) and Q (W239-A259), form the first of three TPR-motifs and are 9 residues longer than the eponymous 34 residue-long tetratricopeptide motif. In the second TPR motif formed by helix R (D262-K285) and S (W289-L300), helix R is again extended by an additional helical turn of four residues compared to a canonical TPR motif. An insertion in this location is a feature that distinguishes the immunophilin TPR domains from TPR domains present in the larger group of chaperone proteins that compete for Hsp70 or Hsp90 with Cyp40. The third TPR motif formed by helices T (T307-G319) and U (Y323-I336) conforms to the canonical repeat length of 34 amino acids. The 7th helix V (K341-K362) caps the domain. A well-defined positively charged groove formed by residues on helices P, R, and T provides the binding site for the C-terminal Hsp90 peptide. Site point mutations identified five residues (Lys-227, Asn-231, Asn-278, Lys-308, and Arg-312) lining the groove as essential for Hsp90 binding.^8^ Deletion or mutation of the Hsp90 carboxyl terminus (729MEEVD732) completely abrogates Cyp40 binding.^9^

In an earlier paper,^10^ we showed that binding of a heat shock peptide at the TPR domain allosterically affected enzyme activity of the PPIase domain. Comparison of molecular dynamics (MD) simulations for the apo and a holo peptide (MEEVD) bound state of Cyp40 showed a significant reduction in root mean square (RMS) fluctuation in both TPR and cyclophilin domains when MEEVD is bound. The MD simulations of the apo protein highlight strong anti-correlated motions between residues around the PPIase-active site and a band of residues running across four of the seven helices in the TPR domain which were significantly reduced in the holo structure explaining the loss of PPIase activity upon peptide binding.

The TPR domain protein carboxy-terminus of Hsc70 interacting protein (CHIP, PDB code 2C2L)^11^ is an E3 ligase that binds to the C-terminal EEVD motif in a very similar manner to that of the Cyp40-Hsp90 complex. Biochemical and biophysical studies demonstrated that mutations or peptide binding to the TPR domain had a significant allosteric effect on the E3-ligase activity as measured by ubiquitination of substrate protein p53. The CHIP structure (PDB code 2C2L) binds the Hsp70 which shares the same carboxy terminal EEVD motif as Hsp90. The peptide binds in a positively charged groove with similar topology and charge distribution as the binding groove in the Cyp40-Hsp90 complex. Mutation of a lysine residue K30A in the binding groove of the CHIP TPR domain abrogates Hsp70 binding and significantly reduces E3 ligase activity. The E3 ligase active site is over 40 Å away from the K30A mutation and suggests a clear allosteric effect.^11^

The observation that allosteric effects can be caused by site-point mutations in a TPR domain remote from the active site suggests that similar allosteric effects on the enzyme activity of the PPIase domain of Cyp40 could be engineered by means of site-point mutations of its TPR domain. Of particular interest are the carboxylate clamp residues Lys227 and Lys308 which form salt bridges with the MEEVD C-terminal residues of Hsp90 (Fig. 1). In this paper we describe MD simulations of three mutations in the TPR domain: K227A, K308A, and the double mutant K227A + K308A. These three proteins have also been expressed and purified and biophysical measurements including Differential Scanning Calorimetry (DSC) data provide experimental data to interpret the MD simulations. A total of 5.76 *μ*s of all-atom MD simulations were performed for the (i) wild type (WT), (ii) K227A mutant, (iii) K308A mutant, and (iv) K227A + K308A double mutant.

## II. MATERIALS AND METHODS

### A. Molecular dynamics system preparation

The cyclophilin 40 (Cyp40) structure having PDB ID: 1IHG^6^ was selected for simulations/computations. The bound ligand (GOL) was removed to obtain an apo WT structure of Cyp40. Three mutants were generated starting from this WT Cyp40 structure: (i) CY227, where residue Lys227 was mutated to alanine (K227A), (ii) CY308, where lysine 308 was mutated to alanine (K308A), and (iii) CYD, where both lysine 227 and 308 were mutated to alanine (K227A, K308A). These mutations were performed in VMD^12^ using the Mutator Plugin, Version 1.3. Similarly, all remaining steps for system preparations were performed in VMD. Each structure was solvated in a water box having at least a 12 Å cushion of water in each direction from the exposed atoms. Ions were added to neutralize the system and set the NaCl concentration to 150 mM. The sizes of the solvated wild type (WT), K227A, K308A, and double-mutant systems were 53 783, 53 772, 53 790, and 53 779 atoms, respectively. Simulations were performed in NAMD 2.10^13^ (with NVIDIA CUDA acceleration) using the CHARMM36 force field.^14^ A cutoff distance of 12 Å was adopted for van der Waals (vdW) interactions, with a switching function starting at 10 Å and reaching zero at 12 Å. Long-range electrostatic forces were computed using the particle-mesh Ewald method. A time step of 2 fs was used. Simulations were performed under NPT ensemble conditions; temperature and pressure were kept constant at 310 K and 1 atm, respectively. A damping coefficient of 1 ps^−1^ for Langevin dynamics was used to maintain isothermal conditions. The Langevin Nosé-Hoover method was performed using an oscillation period of 100 fs and a damping time scale of 50 fs to maintain the pressure constant. First, 10 000 steps of minimization followed by 2 ns of equilibration was performed by keeping the protein fixed. Subsequently, the complete system was minimized for additional 10 000 steps without any constrains/restrains on the protein. The resulting conformer was equilibrated for 6 ns; where, during the first 2 ns of simulations, harmonic constraints having force constants of k = 2 kcal mol^−1^ Å^−2^ were applied to the backbone atoms. Each system was subsequently simulated for 720 ns (production run, please see Table I for details) and conformations were recorded every 50 ps; hence ending up with 14 400 conformers for each system.

**TABLE I.** Simulation conditions and durations of the runs performed.

MD simulation details								
Simulation index	MD1	MD2	MD3	MD4	MD5	MD6	MD7	MD8
Mutation	WT	WT	K227A	K227A	K308A	K308A	K227A	K227A
							K308A	K308A
Length (ns)	720	720	720	720	720	720	720	720

### B. Principal component analysis

All conformers in the WT, K227A mutant, K308A mutant, and K227A + K308A double mutant were aligned with the crystal structure using the C^α^ atoms. The 4 residues at the N-terminal and C-terminal ends were not included in the alignment due to their high degree of fluctuations. After alignment/superposition, the covariance matrix C was constructed^15^ as follows:

$$C=\left\langle \left(R-\left\langle R\right\rangle \right){\left(R-\left\langle R\right\rangle \right)}^{\text{T}}\right\rangle .$$ (1)

**R** is the 3 × 364-dimensional configuration vector composed of the instantaneous C^α^-atom coordinates of residues S2-Y365 and $\left\langle R\right\rangle$ is the trajectory average of **R**. Principal components (PCs) are constructed by performing eigenvalue decomposition of the covariance matrix,

$$C=\sum_{i=1}^{3N}\sigma_{i}p_{i}p_{i}^{T}.$$ (2)

Here $p_{i}$ is the *i*th PC having a variance of $\sigma_{i}$. PCs are ordered in descending order of their variance values. Essentially, PC1 has the largest variance and the vector denotes the most dominant motion/feature observed in the MD simulations.

### C. Calculation of correlation matrices and B-factors

Correlations between the motions of subsets of atoms can be extracted from the elements of the covariance matrix shown in Eq. (1). When the index i is equal to j, we obtain the atomic mean square fluctuation (MSF), ⟨(**ΔR**_**i**_)^2^⟩. The crystallographic temperature factor, or B-factor, is related to the MSF by ⟨(**ΔR**_**i**_)^2^⟩ = (3/8π^2^)B_i_.

### D. Angle between helices

Principal component analysis (PCA) is performed on all C^α^ atom coordinates of a helix. For each helix PC1 vectors taken, $p_{1}$ and $l_{1}$ and their dot and cross products are appointed to x and y variables as $x=l_{1}\cdot p_{1}$ and $y=l_{1}\times p_{1}$, respectively. The angle between $p_{1}$ and $l_{1}$ is calculated as θ=arctan(y,x), which is the angle in radians between the positive x-axis of a plane and the point given by the coordinates (x,y).

### E. Protein production

The open reading frame for human Cyp40 was synthesized with a six histidine tag and a protease cleavage site, TEV, added at the N-terminus (MSKY**HHHHHH**DYDIPTTENLYFQG-Cyp40) (GeneArt™). Standard GATEWAY® methodology was used to generate an expression vector in pDEST™14 (Thermo Fisher, Waltham, MA, USA). Lysine to alanine mutants K227A, K308A and the double mutant K227A + K308A were introduced using the GeneArt Site-Directed Mutagenesis System (Thermo Fisher). Proteins were overexpressed in *One Shot*® BL21 Star™ (DE3) Chemically Competent cells (Thermo Fisher) as described in the work of Wear *et al.*^16^ In brief, proteins were first captured by ion-metal affinity chromatography (1 ml, HiTrap™ IMAC, ff; GE Healthcare) and then further purified to homogeneity by size-exclusion chromatography (Superdex™ 200 10/300 GL; GE Healthcare). Purity was assessed to be greater than 95% by SDS/PAGE.

### F. Differential scanning calorimetry (DSC)

Differential scanning calorimetry experiments were performed on a MicroCal VP-capillary DSC system (GE Healthcare). Proteins were exchanged into 50 mM HEPES, pH8, 150 mM sodium chloride, 1 mM DTT to a final concentration of 13.7 *μ*M and degassed prior to analysis (HiTrap Desalting, 5 ml; GE Healthcare). Cyp40 was heated from 5 to 85 °C at a scan rate of 60 °C/h after a 5 min pre-scan equilibration. All samples were collected in triplicate. Data were normalized for concentration and baseline corrected by subtracting a buffer scan collected under the same conditions as the protein samples. Averaged data were analyzed to determine the mid-point melting temperature, T_m_, and the calorimetric enthalpy, ΔH, using the software provided by the manufacturer (Origin, 7.0).

### G. Peptidyl-prolyl isomerase activity

The PPIase activities of WT Cyp40, K227A, K308A, and their combinative mutant K227A + K308A were compared by determining the rate of the *cis* to *trans* conversion of the substrate Suc-Ala-Leu-Pro-Phe-p-NA (Sigma) at 5 °C, as previously described by Wear *et al.*^17^ using the method originally reported by Kofron.^18^ The final solution contained 20 nM Cyp40; 0.6 mg ml^−1^ α-chymotrypsin; 120 *μ*M Suc-Ala-Leu-Pro-Phe-*p*-nitroaniline; 50 mM HEPES, pH8; 100 mM sodium chloride 14 *μ*M lithium chloride; 1 mM DTT; 0.5 mM EDTA; and 3% 2,2,2-trifluoroethanol (v/v). The turnover was followed by measuring absorbance of the solution at 400 nM; this was recorded every 0.1 s for 120 s on a JascoV550 Ultraviolet Visible (UV/VIS) spectrophotometer (ε_400nm_ = 10 050 M^−1^ cm^−1^; *p*-nitroaniline). Protein concentration was determined from absorbance at 280 nm (ε_280nm_ = 32 890 M^−1^ cm^−1^). Each experiment was repeated at least 6 times and results were reported as mean and standard errors.

## III. RESULTS AND DISCUSSION

### A. Cyp40 mutations stabilise two distinct conformational states

**FIG. 2.**
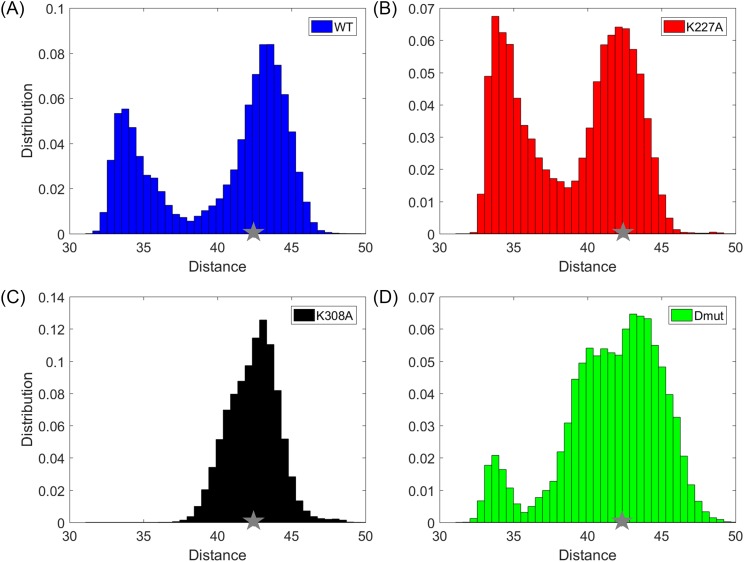
Distribution of the distance between cyclophilin and TPR domains observed in the MD simulations. The normalized distribution of the inter-domain distances between the cyclophilin and TPR domains is shown for the (a) WT (blue), (b) K227A mutant (red), (c) K308A mutant (black), and (d) K227A + K308A double mutant (green). The inter-domain distance is calculated as the distance between the arithmetic center of all C^α^ atoms of the PPIase domain (1–183) and TPR domain (V216- K362). The crystal structure is shown with a gray star.

The interdomain distance between the centres of the TPR and cyclophilin domains as measured in the crystal structure is 41 Å. Over the course of 1.44 *μ*s (2 × 0.72 *μ*s), the MD simulations show significant deviations from this value, Fig. 2: WT Cyp-40 demonstrates a clearly bimodal distribution with a peak corresponding to a more highly populated extended form at 43.8 Å and a less populated compact form at 34 Å. A similar bimodal distribution is observed for the K227A but in this case with the compact form peak at 34 Å similarly populated with the extended form peak centred on 42.1 Å. By contrast, the K308A mutant has only one conformer family with a single peak centred at 43.3 Å suggesting a stabilizing effect of this mutation for the more extended conformation. The K227A + K308A double mutant shows rather broad distribution with two overlapping distributions centered on 40.1 and 43.1 Å and a smaller peak at 33.8 Å; hence, the “extending” effect of K308A seems to be larger than the “compacting” effect of K227A.

**FIG. 3.**
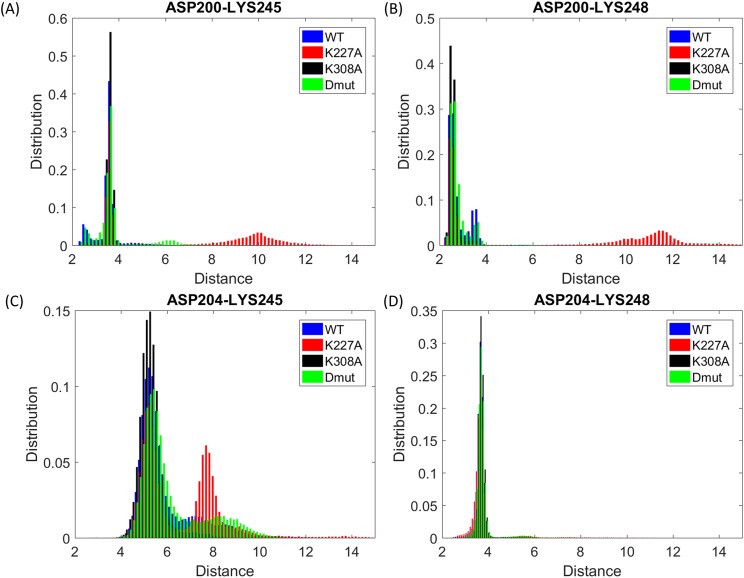
Distribution of the distance between selected linker-region illustrating the potential for salt-bridge formation. The distribution of the distance between the center of mass of the oxygens in the acidic side chain and center of mass of the nitrogens in the basic side chain between the specified amino acids is shown for the WT (blue), K227A mutant (red), K308A mutant (black), and the K227A + K308A double mutant (green).

The different conformations have also been analyzed by monitoring salt bridge distances of residues in the linker region (L184-L213) that stabilizes and regulates the relative movements of the cyclophilin and TPR domains. In the crystal structure, the linker region (Fig. 1) forms a number of strong salt bridges with the TPR residues, the most important being Asp200_linker_–Lys245_helixQ_, Asp200_linker_–Lys248_helixQ_, and Asp204_linker_–Lys248_helixQ_. These salt bridges have been monitored over the length of the simulation by measuring the distance between the center of mass of the oxygens in the acidic side chain and the basic nitrogens (Fig. 3). The distributions of these distances show that the “extended” form adopted in the crystal and favoured by the K308A mutation maintains these salt bridges; they are less well preserved by the K227A mutant. The structures of the extended and compact forms are presented in Fig. S1 of the supplementary material. The extended-form stabilizing effect of the K308A mutation is further supported by significantly lower linker B-factors observed for the K308A mutant, as shown in Figs. S2 and S5 of the supplementary material. In the extended form, the linker domain spatially places itself between the cyclophilin domain and helix Q of the TPR domain, hence strongly limiting TPR-cyclophilin domain interactions. In the compact form, the linker appears to pull back to some degree from the space between these two domains, breaking the salt bridges with Asp200 and Lys248 and allowing TPR and Cyp40 domains to come to a closer relative position (Fig. S1 of the supplementary material).

### B. Domain movements are significantly altered by site-point mutations

#### 1. Principal component analysis of WT and mutant Cyp40

**FIG. 4.**
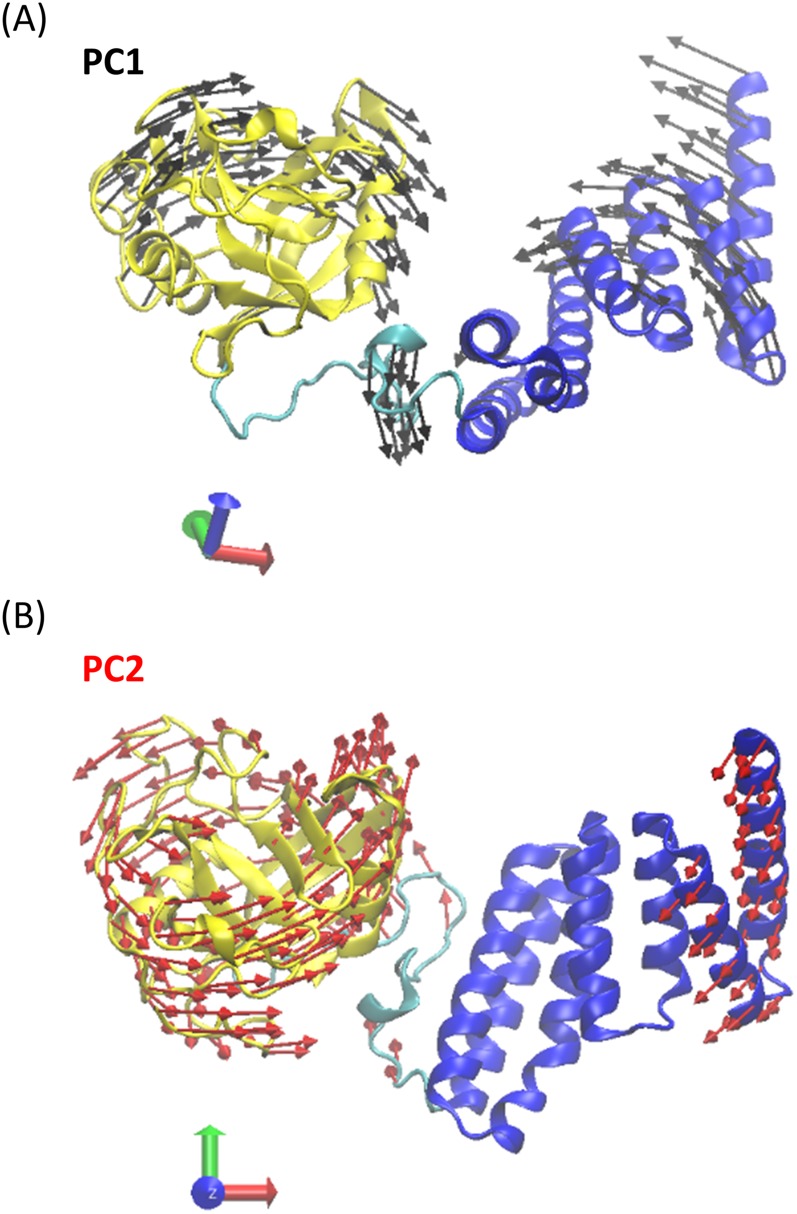
The first two principal components of the complete Cyp40 MD data superimposed on the crystal structure. Simulation data for WT, K227A, K308A, and K227A + K308A Cyp40 were combined into a single trajectory. All conformers were superimposed with the crystal structure based on the alpha carbon atoms. For visual purposes, PC Vectors are shown as superimposed/placed on the crystal structure.

To understand large scale dominant motions, we performed a principal component analysis (PCA) on all conformers of the WT, K227A mutant, K308A mutant, and K227A + K308A double mutant MD simulations. The principal component analysis is an effective and proven method to reveal the most prominent motions a protein exhibits along its MD trajectory, details of which are explained in Sec. II. PC1 and PC2 represent the dominant and the second most dominant motion observed in the MD simulations, respectively. As can be seen in Fig. 4, PC1 is a mode of motion that moves the cyclophilin and TPR domain towards each other. It also moves the linker out from the space between the TPR and Cyp40 domains, making space for the compact form. It seems therefore that the transition from the extended to the compact form is effectively described by the PC1 vector. The second most dominant motion PC2 describes a different set of motions in which the cyclophilin domain rotates around its axis and helices U (Y323-I336) and V (K341-K362) move in a concerted fashion as shown in Fig. 4(b).

**FIG. 5.**
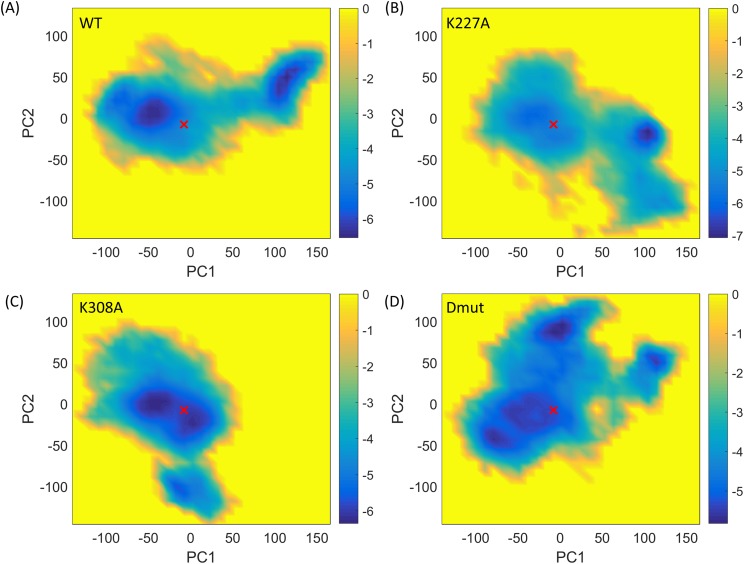
Energy landscape for Cyp40. Energy landscape generated for (a) WT, (b) K227A, (c) K308A, and (d) K227A + K308A Cyp40 simulations. Reaction coordinates defining the x and y axis was defined as the principal components obtained by combining all sets of simulations. The crystal structure is shown with a red cross. Higher positive values along PC1 indicate an increased degree of compactness of the MD conformers, whereas lower negative values indicate an elevated separation/extension between the TPR and Cyp40 domains.

**FIG. 6.**
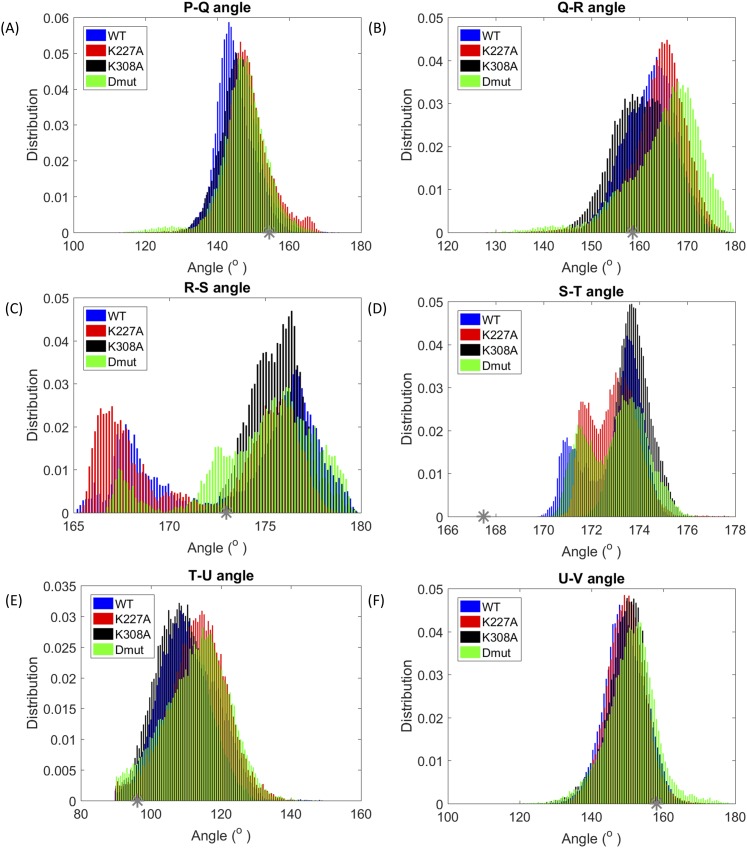
Distribution of the angles between TPR helices. The normalized distribution of the angle between the TPR helices is shown for the WT (blue), K227A mutant (red), K308A mutant (black), and the K227A + K308A double mutant (green). The angles corresponding to the crystal structure are shown with gray stars.

Subsequent to the PCA, MD snapshots were projected onto the principal components (PCs). The distributions of the projections, f(R), were computed along sets of PCs. Higher positive values along PC1 indicate an increased degree of compactness of the MD conformers, whereas lower negative values indicate an elevated separation/extension between the TPR and Cyp40 domains. Using these distributions, free energy surfaces were calculated as $A\left(R\right)=-kT\ln\left(f\left(R\right)\right)+\text{constant}$.^19^ The free energy surfaces of the WT, K227A mutant, K308A mutant, and K227A + K308A Cyp40 projected on the principal components (PCs) 1-2 are shown in Fig. 5. As can be clearly seen in Fig. 4, along PC1 the K308A mutant samples regions which are predominantly close to the crystal structure (shown with a red cross), whereas for the K227A mutant there is a clear alternative energy minimum probably corresponding to the “compact” conformational form. Hence, K227A is more likely to exhibit the motions associated with PC1. This observation is in accord with our previous study where we have shown that a highly localized mode of motion can energetically couple and hence activate/excite a global mode of motion.^20^ The differences in the features of the free energy surfaces strongly support the observation from monitoring inter-domain distances (Fig. 2) that the K308A mutation stabilizes the “extended” conformation favoured by the crystal structure. As it is implied from the different distributions of each system along the PCs, there are some significant differences in domain motions between the different point mutant structures, in particular, the angles between the consecutive helices in the TPR domain (Fig. 6).

#### 2. The TPR domains of the mutants show distinctly different motions

The TPR domain has very few direct H-bonds between residues maintaining the fold after Lys227_helixP_-Ser274_helixQ_. Hydrophobic interactions between helices, particularly the last 5 amino acids E_361_KAAY_365_ on one TPR and the groove of another, provide stability for the fold in the crystal structure. When Cyp40 is in complex with Hsp90, additional non-covalent interactions are provided by binding MEEVD. Mutating K227A uncouples the two halves of the TPR domain.

As shown in Fig. 6, the angle between helices R and S shows a two-peak distribution for WT and K227A and a single peak distribution for the K308A mutant. Similarly, for the S-T angle, a two-peak distribution was observed for WT, K227A, and Dmut and a single peak was observed for K308A.

#### 3. Pairwise distance analysis also highlights differences in TPR movement

The distance between residues i and j is calculated from the trajectory as follows: the distance between the alpha carbons of residues i and j is calculated for all residue pairs at each time station of the trajectory and the average is obtained by summing up these values and dividing by the number of time stations.

**FIG. 7.**
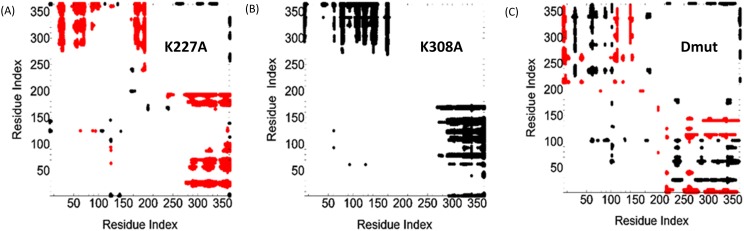
Changes in residue pair distances upon mutation. The abscissa and the ordinate identify the residue indices. A red point on the graph corresponding to a residue i and a residue j shows that the distance between those two residues is decreased upon mutation. A black point indicates an increase. The red points show decreases between −10 and −4 Å. Black points show increases between 4 and 10 Å. Panel (a) shows the distance changes for the system K227A, panel (b) is for K308A, and panel (c) is for the double mutant, K227A + K308A.

Changes in the distance, *d*_ij_, between two residues i and j upon mutation are presented in Fig. 7. A decrease in *d*_ij_ upon mutation is denoted by a red point in the figure, and an increase is denoted by a black point. Red regions show decrease in distance, between −10 and −4 Å. Black regions show increase between 4 and 10 Å. This suggests that K227A Cyp40 has an average conformation that brings the last four helices (S, T, U, V) of the TPR closer to the Cyp40 domain, while the K308A mutation has the opposite effect of keeping domains apart. Interestingly this divergence involves the residues of the PPIase domain, 50-160, that are not in close proximity to the linker. In the double mutant, the situation is more complex; the active site face of the cyclophilin domain twists away from the TPR domain but the anterior face moves closer. This is shown in the red and black stripes for the double mutant in Fig. 7.

#### 4. Changes in pair correlations upon mutation

**FIG. 8.**
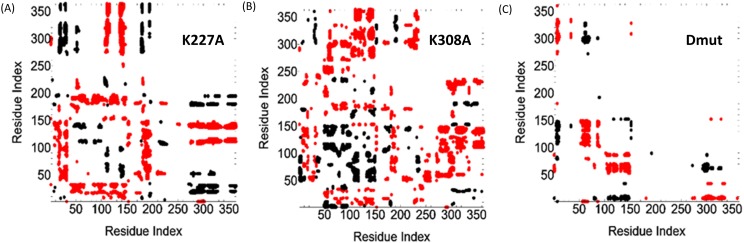
Changes in negative correlations upon mutation of the wild type. The abscissa and the ordinate identify the residue indices. The red and black points show anticorrelated residues. A red point shows that residues i and j have become less anticorrelated upon mutation. A black point shows that residues i and j have become more anticorrelated upon mutation. Panel (a) shows the changes for the system K227A, panel (b) is for K308A, and panel (c) is for the double mutant, K227A + K308A.

Correlation matrices for each of the mutants were calculated (see Sec. II). Figure 8 shows differences between correlations observed in the WT and those observed in the three mutants. Points corresponding to anticorrelations are only shown. Red points denote a decrease in anticorrelations and black points show an increase. For the single mutants, there is a marked loss of anticorrelated motions (red points) between PPIase and TPR domains, while the double mutant shows marked loss (red points) of anticorrelated motion between residues 105 and 150, residues that form the boundary of the active site.

The pattern of increased anticorrelations (black points) of the C-terminal helices T, U, V, W (residues 285-380) fits an exaggerated wagging motion of the last four helices which will be both enhanced negative and positive correlations with different groups of residues on the rotating cyclophilin (Fig. 6). This explains the interesting red and black striped pattern for K227A Cyp40 (Fig. 8). Black stripes (TPR residues 290–362) represent an enhanced anti-correlation with the N-terminal and C-terminal cyclophilin residues 1-50_PPIase_ and 180-210_linker_, while red stripes highlight a decreased anticorrelation compared to WT Cyp40.

#### 5. Melting temperature and protein compactness are correlated

The MD simulations have been used to identify and quantify two different measures of conformational compactness each of which correlates with the melting temperature. Mid-point melting temperatures (T_m_) have been measured for WT Cyp40 and each of the mutants as described in Sec. II. A summary of the results is given in Table II.

**FIG. 9.**
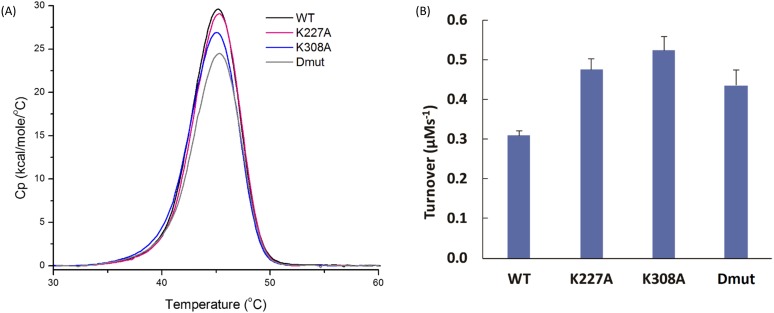
Normalized specific heat (Cp) endotherms and PPIase enzyme activity. (a) Normalised specific heat capacity (Cp) endotherms measured by Differential Scanning Calorimetry (DSC) for WTCyp40, K227ACyp40, K308ACyp40, and K227A/K308ACyp40. DSC endotherms for the melting of WTCyp40 (black), K227ACyp40 (magenta), K308ACyp40 (blue), and K227A/K308ACyp40 (gray) show mutating lysine 227 and lysine 308 reduces the enthalpy of unfolding of Cyp40. The calorimetric enthalpy, ΔH, corresponds to the area under the curve. Contributions of site point mutants to the enthalpy of unfolding are additive (see Table II for values). Samples were scanned from 5 to 85 °C at a scan rate of 60 °C/h after a 5-min pre-scan equilibration in 50 mM HEPES, pH8, 150 mM sodium chloride, and 1 mM DTT. Data were baseline corrected by subtracting a buffer scan collected under the same conditions as the protein samples and normalized with respect to protein concentration. Thermodynamic parameters were determined using the software provided by the manufacturer (Origin, 7.0). All data were collected in triplicate. (b) Mutation of lysine 227 and lysine 308 enhance PPIase enzyme activity. Comparison of the biochemical activity of WTCyp40, K227ACyp40, K308ACyp40, and K227A/K308ACyp40. Cyp40 catalyses the *cis-trans* isomerisation of the tetrapeptide peptide substrate s-ALPF-*p*-nitroaniline. Mutating lysine 227 and lysine 308 enhances the activity of Cyp40. The final solution contained 20 nM Cyp40; 0.6 mg ml^−1^ α-chymotrypsin; 120 *μ*M s-ALPF-*p*-nitroaniline; 50 mM HEPES, pH8; 100 mM sodium chloride 14 *μ*M lithium chloride; 1 mM DTT; 0.5 mM EDTA; 3% 2,2,2-trifluoroethanol (v/v). Enzyme catalysed turnover has been corrected for the thermal turnover of the substrate. Data represent the mean of 6 replicates with the standard error.

**TABLE II.** Characterisation of WT Cyp40 and three mutants. R_g_ is the radius of coordination shell volume T_m_ is the melting temperature, ΔH is the normalized calorimetric enthalpy, the area under the curve of the C_p_ endotherm (Fig. 9), turnover is catalytic activity, and % extended is an estimate from conformer distribution.

	Average residue		ΔH_cal_	Turnover	% protein in
	R_g_ (Å)	T_m_ (^°^C)	(kcal/mol)	(*μ*M s^−1^)	compact form
WT	6.64	45.10	164.6	0.31	30
K308A	6.60	45.03	154.6	0.52	0
K227A	6.71	45.22	159.1	0.47	60
Dmut	6.72	45.30	138.3	0.44	50

#### 6. Coordination shell volume provides a measure of protein compactness and correlates with T_m_

**FIG. 10.**
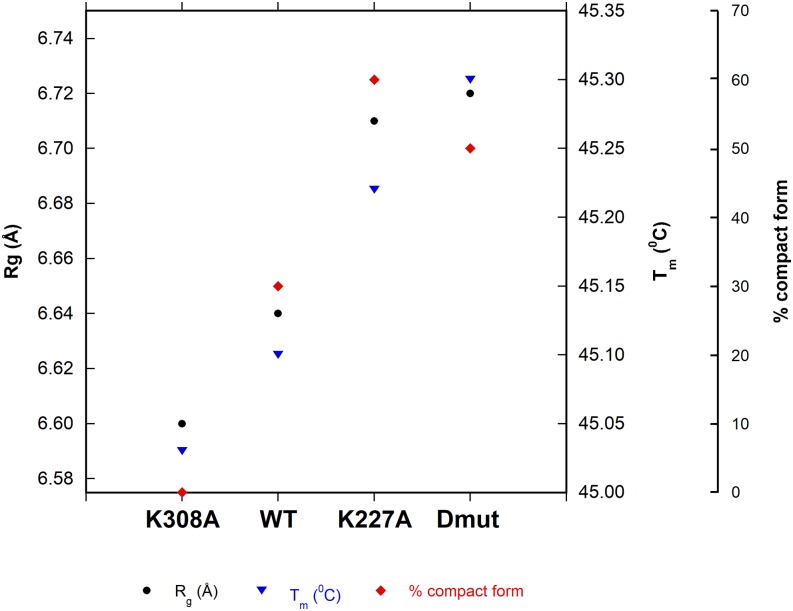
Residue radius of coordination shell volume correlates with the proportion of protein in the compact form and T_m_. The figure shows a correlation between R_g_ (a metric that describes the average radius of coordination shell volume of each residue derived from the MD simulation) with the percentage of the protein in the compact form (calculated by considering the distance between the geometric centre of the PPIase domain and the TPR domain over the simulation) and the experimentally derived mid-point melting temperature, T_m_ (see Sec. III B).

As a measure of the compaction effect of the mutations, we developed a parameter defined as “coordination shell volume.” The radius of coordination shell volume is estimated as follows: The C^α^ of residue i is chosen as the center and the distances to the neighboring alpha carbons of the residues within the center are calculated. The radius of coordination shell volume is calculated as $R_{g}={\left(\sum R_{ij}^{2}/n_{i}\right)}^{1/2}$. $n_{i}$ is the number of neighbours to the ith C^α^ atom. The radii are averaged over all residues of the protein. Figure 10 shows that single point mutations change the size of the coordination shell over both domains of the protein with R_g_ values that range from 6.60 Å (K308A) to 6.72 Å (Dmut). They show an interesting correlation with melting temperature as determined using differential scanning calorimetry (Fig. 9 and Table II). The differences in T_m_ between WT and mutants are small with a maximum decrease or increase in T_m_ of 0.2^0^ (K308A 45.03, WT 45.10, K227A 45.22, Dmut 45.30).

#### 7. The folded and extended states of Cyp40 correlate with T_m_

As described above, the MD simulation identifies extended and compact conformations of Cyp40 which are measured by the distance between the centroids of the TPR and Cyp domains. The most extended state is adopted by K308A which has an interdomain distance of 43.3 Å (0% folded) and the most compact adopted all other forms at close to 34.0 Å; the size of this population varies. The “foldedness values” (or molecular compactness) also correlate with the measured T_m_ values, Fig. 10.

### C. Differences of calculated B-factors for wild type and mutant Cyp40 may explain differences in PPIase activities

**FIG. 11.**
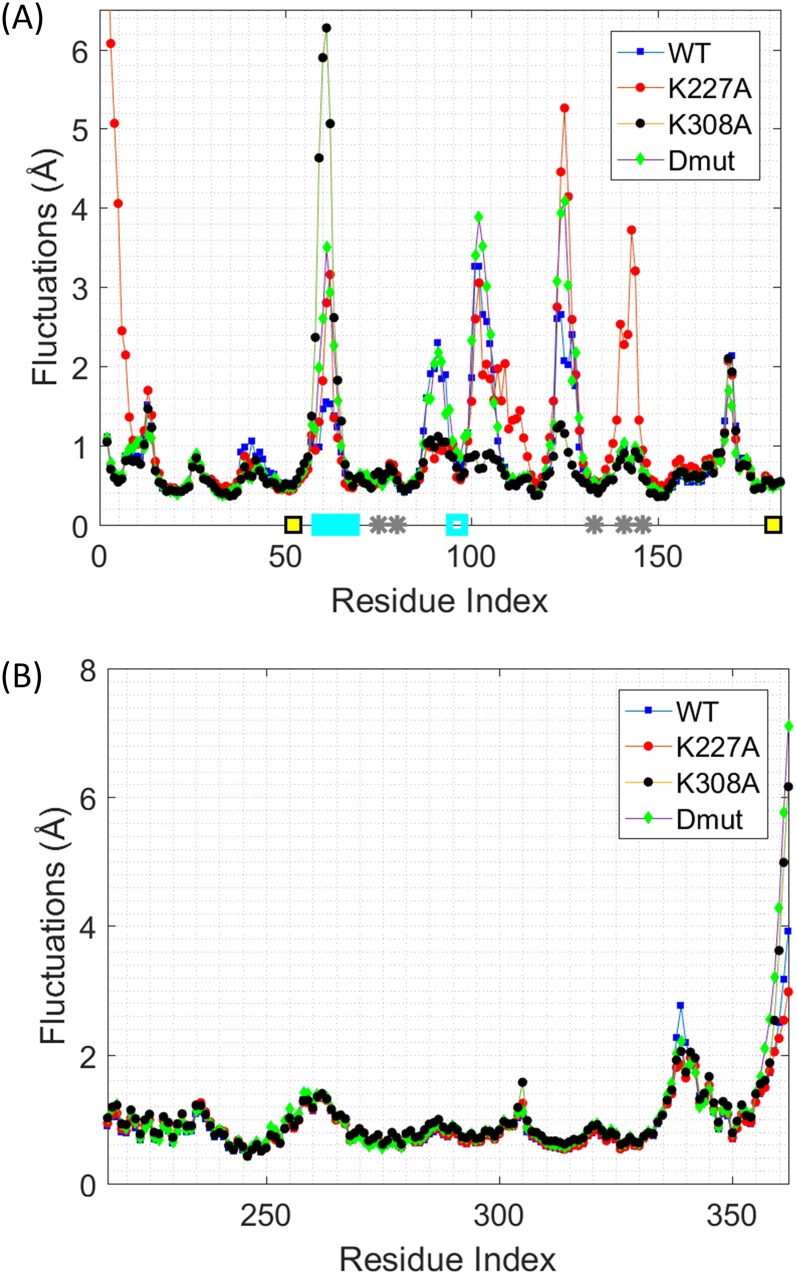
Residue fluctuations (B-factors) of the cyclophilin and TPR domains observed in the MD simulations. Average residue fluctuations are shown for the (a) cyclophilin and (b) TPR domains for the WT (blue), K227A mutant (red), K308A mutant (black), and the K227A + K308A double mutant (green). Residue fluctuations are calculated for each residue *i* using its C^α^ atom fluctuation via ${\left\langle \left(R_{i}-\left\langle R_{i}\right\rangle \right)\cdot \left(R_{i}-\left\langle R_{i}\right\rangle \right)\right\rangle }^{0.5}$. Here $\left\langle R_{i}\right\rangle$ is the MD simulation average of the *i*th C^α^ atom coordinate. The cyan bar shows the location of the divergent loop. Positions of active site residues are shown with gray stars. Yellow squares show the positions of the cysteines.

The intradomain correlation matrices were separately calculated from the average fluctuation vectors for each C^α^ atom in the cyclophilin and TPR domains of WT Cyp40, K227A Cyp40, K308A Cyp40, and the double mutant (Dmut) (see Sec. II). For example, to obtain the TPR domain correlation matrices of Cyp40 (WT), all Cyp40 conformers in the MD simulations were aligned with respect to their TPR domains and a correlation matrix was constructed using the C^α^ atom coordinates of the aligned TPR domain. The diagonal terms for each matrix are used to calculate residue fluctuations (B-factors) shown in Fig. 11. Intriguingly the biggest fluctuations (and differences between mutants) occur in the cyclophilin domains which are remote from the site of the mutations which occur on the TPR domains. It is also surprising that there are only small changes in flexibility (temperature factor) in the mutated TPR structures (around residue 308 where the K308A mutation has a higher B-factor) and around 340 where all three mutations have lower B-factors.

B-factors were calculated separately for cyclophilin, TPR (Fig. 11), and linker domains (Fig. S2 of the supplementary material) domains. Domain average B-factors were calculated as $\left[\sum {\left\langle \left(R_{i}-\left\langle R_{i}\right\rangle \right)\cdot \left(R_{i}-\left\langle R_{i}\right\rangle \right)\right\rangle }^{0.5}\right]/n$, where *n* is the number of residues in each domain, and are show in Table 1 of the supplementary material. The domain average B-factor of the linker domain was significantly higher than those for the cyclophilin (Å) and TPR domains (Å). For the WT, the domain average B-factor of the linker was 3.1 and 3.3 times as much as the cyclophilin and TPR domains, respectively. Similarly, for the K227A mutant, the linker domain average B-factor was 2 and 2.5 times as much as the cyclophilin and TPR domains. For the Dmut simulations, average linker domain fluctuations were 2.4 and 2.1 times as much as the cyclophilin and TPR domains. For the K308A mutant, on the other hand, ratios were much smaller; the linker domain fluctuated on average only 1.7 and 1.3 times as much as the cyclophilin and TPR domains.

Experimentally we have shown that all forms of Cyp40 were enzymatically active. The measured enzyme activities for the three mutants all show a small increase in turnover rate compared with WT Cyp40: WT, 0.31 *μ*M s^−1^, Dmut 0.44 *μ*M s^−1^, K227A 0.47 *μ*M s^−1^, and K308A 0.52 *μ*M s^−1^[Fig. 9(b)], which shows that the Lys to Ala mutations on the TPR domain have an allosteric effect on the cyclophilin active site of the protein.

Although we have not yet identified a specific mechanism explaining the enhanced enzyme activity caused by the lysine mutations in the remote TPR domain, it is interesting to note that the all three mutations do have a clear effect on the fluctuations of specific regions of the cyclophilin domain. The most prominent effect is the increased fluctuation in the divergent loop (60PTTGKPLH67) which may enhance substrate recognition. This effect is most pronounced for K308A Cyp40. Also interesting is an increase in fluctuation of the catalytic residues H141 and H146 observed in the K227A mutant.

### D. Contributions of site point mutants to the enthalpy of unfolding are additive

ΔH is the normalized calorimetric enthalpy of un-folding (ΔH), measured as the area under the curve of the C_p_ endotherm. It is significantly different for each of the mutants. ΔH provides an absolute measurement of the heat energy uptake and it is notable that WT has a significantly higher value (165 kcal/mol) compared to ΔH for Dmut (138 kcal/mol). The two single K to A mutants have intermediate values. This suggests that unfolding energy is related to the number of free lysines which are likely to form a strong network of hydrogen-bonded interactions with solvent. The difference in the melting enthalpy between WT and the three mutants are ΔΔH K227A = 5.5 kcal/mol, ΔΔH K308A = 10.0 kcal/mol, and ΔΔH Dmut = 16.3 kcal/mol. To a first approximation it would seem that the individual contributions of the single point mutations are additive with a Dmut value of 16.3 kcal/mol close to 15.5 kcal/mol, the combined values of the individual mutants. Reducing the hydrogen bonding network for the double mutant Cyp40 lowers the overall stiffness of the protein thus increasing entropy. This is a feature of thermophilic proteins which enables them to function at higher temperatures:^21^ it has the effect of enabling the protein to explore more conformers, thereby increasing the melting temperature compared to WT; Table I and Fig. 5.

In Fig. 10, the effect of mutation on the “coordination shell volume” (MD simulations), molecular compactness (MD simulations), and melting temperatures (experiments), are shown. There is a clear change in parameters for each single mutant. Interestingly, neither a simple addition nor taking averages of singe mutations K308A and K227A result in the values obtained for double mutant. Double mutant values clearly tend to be closer to the K227A mutant.

## IV. CONCLUSION

The long simulation times of the WT and the three mutants show clear differences in the domain movements caused by site point mutations. K308A favours the stretched form with 43 Å separation between the cyclophilin and TPR domains distinguish stretched and compressed forms, while K227 about 50% of the conformers adopt a compressed form with a separation of 34 Å. These two forms can be rationalised by the domain motions identified by principal component analysis (Fig. 4) which, especially for PC1, shows correlated movements that combine to move the two domains closer together.

Similarly the analysis of the helical angles fits with the movements identified in PC2. In particular the large change in the angle between helix R and S which is very pronounced for K227A (Fig. 6) is recapitulated in the movement of the final three helices (T, U, and V) identified in PC2 (Fig. 6). The X-ray structure of the apo protein shows a strong hydrogen bond between K227_helixP_ and S274_helixR_ tying together helices P and R. The K227A mutation will relax that constraint and is consistent with the observation that about 50% of the K227A conformers adopt a compressed form with a separation of 34A. By contrast, K308A only adopts the extended form. In the X-ray structure, K308 points out into solvent, forming crystal contact with the C-terminal stretch of its neighboring Cyp40, and mutation to alanine can not directly affect interhelical contacts. However the intrinsic helical stabilisation of alanine^22^ and related changes in the solvent structure act to constrain this region by allowing slightly closer packing between helix S and T of the TPR domain and maintain the extended form. The changes in salt-bridge formation at the linker region are also consistent with the different allosteric effects of the K227A and K308A mutations. The key stabilising salt bridges (D200-K245, D200-D248, and D204-K245, Fig. 3) are all broken in the compact conformer favoured by K227A but retained by the K308A mutant. In our earlier study,^10^ we showed that MEEVD binding resulted in smaller fluctuations compared to the apo WT form. A similar effect was observed in our simulations for K308A. As can be seen in Fig. 1 upper middle panel, K308 is a residue directly interacting with the MEEVD peptide. Hence, it may be concluded that K308A mutation mimics to some extend the effect of substrate binding.

The E3 ligase CHIP has a similar TPR architecture to that of Cyp40 and a single Lys-Ala mutation resulted in an increase in T_m_ of 3.5^0 11^. In both CHIP and Cyp40, the K to A mutations in the TPR domain have an allosteric effect on the catalytic domain. Three tetratricopeptide motif tandem repeats appear to be inherently flexible due to their packing being largely driven by hydrophobic interactions. The extensive MD simulations of Cyp40 and its mutants presented here show that the mutations can affect distinctly different conformers. The experimentally observed differences in T_m_ correlate with measures of compactness: more extended and less compact structures melt at slightly lower temperatures. Interestingly the mutations in the remote TPR domain have a pronounced effect on the molecular motions of the enzymatic cyclophilin domain which appear to be correlated with a slightly enhanced enzymatic activity for all three mutants.

## SUPPLEMENTARY MATERIAL

See supplementary material for 4 figures. Figure S1 shows the structure of the extended form of cyclophilin 40 in comparison with the conformers in compact form obtained by the WT, K227A, and Dmut MD simulations. Figure S2 displays the B-factors of the linker domain. Figure S3 shows the time evolution of the distance between cyclophilin and TPR domains observed in the MD simulations. Figure S4 depicts the MD conformer distributions along PC1. Figure S5 shows B-factors of the complete protein. Figure S6 shows the crystal structure colored based on the residue fluctuations observed in the MD simulations. In Table S1 the residue fluctuation (B-factor) averages for each domain are provided. Data files providing the means and variances of internal distances and correlations between residues are given.
